# Major Advances in Gynecologic Oncology in 2025: Systematic Review and Synthesis of Conference and Published Evidence

**DOI:** 10.3390/biomedicines14020295

**Published:** 2026-01-28

**Authors:** Nabil Ismaili

**Affiliations:** 1 Department of Medical Oncology, Mohammed VI Faculty of Medicine, Mohammed VI University of Sciences and Health (UM6SS), Casablanca 82403, Morocco; ismailinabil@yahoo.fr or nismaili@um6ss.ma; 2 Mohammed VI Foundation of Sciences and Health (FM6SS), Casablanca 82403, Morocco; 3 Oncopathology, Biology and Environment of Cancer Laboratory, Mohammed VI Center for Research and Innovation (CM6RI), Rabat 11103, Morocco

**Keywords:** gynecologic oncology, immune checkpoint inhibitors, PARP inhibitors, targeted therapy, antibody-drug conjugate, biomarker, clinical trials, ASCO, ESMO

## Abstract

**Background**: The year 2025 witnessed paradigm-shifting advances in gynecologic oncology, with pivotal clinical trial results redefining therapeutic standards across cervical, ovarian, endometrial, and vulvar cancers. **Objectives**: This systematic review aimed to comprehensively identify, synthesize, and critically evaluate pivotal phase II and III randomized controlled trials and major studies presented at the major annual meetings, alongside significant peer-reviewed publications from 2025 that introduce innovative therapeutic strategies across gynecologic malignancies. **Methods**: Conducted in accordance with the Preferred Reporting Items for Systematic Reviews and Meta-Analyses (PRISMA) 2020 guidelines, this review involved exhaustive searches of electronic databases (PubMed/MEDLINE, Embase), conference proceedings (ASCO 2025, ESMO 2025), and major oncology journals for records from January to December 2025. Inclusion criteria encompassed: (1) Phase II or III randomized controlled trials (RCTs) and (2) Non-randomized studies (including phase I and II trials), reporting on novel therapeutic approaches in gynecologic oncology. All studies were required to report primary survival endpoints (overall survival or progression-free survival) or key efficacy outcomes. Study selection, data extraction, and methodological quality assessment were performed independently by two reviewers, with disagreements resolved through consensus or third-party adjudication. **Results**: From 1842 records, 23 studies met inclusion criteria (17 phase-III RCTs and 6 non-phase III RCTs/early-phase studies), distributed as follows: cervical cancer (9 studies, 39%), ovarian cancer (9 studies, 39%), endometrial cancer (4 studies, 17.5%), and vulvar cancer (1 study, 4.5%). The major advances identified include: (1) In cervical cancer, the KEYNOTE-A18 trial established pembrolizumab combined with chemoradiotherapy as a new standard for high-risk locally advanced disease, while the PHENIX trial validated sentinel lymph node biopsy as a safe surgical de-escalation strategy. (2) In ovarian cancer, the ENGOT-ov65/KEYNOTE-B96 trial demonstrated the first statistically significant overall survival improvement with an immune checkpoint inhibitor in platinum-resistant recurrent disease, establishing pembrolizumab plus weekly paclitaxel as a new standard of care. Novel therapeutic mechanisms, including glucocorticoid receptor modulation (ROSELLA trial) and cadherin-6-targeted antibody-drug conjugates (REJOICE-Ovarian01), showed remarkable efficacy. (3) In endometrial cancer, updated analyses from NRG GY018 and RUBY trials solidified the role of first-line immuno-chemotherapy, with differential benefits according to mismatch repair status. (4) In vulvar cancer, a pivotal phase II study demonstrated meaningful clinical activity of anti-PD-1 therapy in advanced disease. (5) The extensive circulating tumor DNA analysis from the CALLA trial provided crucial insights into biomarker dynamics in cervical cancer. **Conclusions**: The convergence of high-impact data from 2025 established multiple new standards of care, emphasizing biomarker-driven approaches, immunotherapy integration across disease stages, and novel mechanisms to overcome resistance, while highlighting challenges in treatment sequencing and global access.

## 1. Introduction

Gynecologic cancers, encompassing malignancies of the ovary, endometrium, cervix, vulva, and vagina, represent a significant global health burden, accounting for approximately 13% of all cancer diagnoses and 10% of cancer-related mortality in women worldwide [1]. The therapeutic landscape for these heterogeneous diseases has historically evolved incrementally, but recent years have witnessed an accelerating pace of discovery driven by advances in molecular biology, tumor immunology, genomics, and targeted drug development. The year 2025, in particular, has proven to be an exceptionally pivotal period, characterized by the presentation of landmark clinical trial results at premier international oncology conferences, most notably the American Society of Clinical Oncology (ASCO) Annual Meeting and the European Society for Medical Oncology (ESMO) Congress, and their concurrent or subsequent publication in high-impact, peer-reviewed medical journals.

This convergence of data dissemination platforms has created a unique and powerful evidence base that is rapidly reshaping clinical paradigms across the spectrum of gynecologic malignancies. From the integration of immunotherapy into curative-intent settings for cervical cancer to the breakthrough of immune checkpoint inhibition in the notoriously treatment-resistant arena of platinum-resistant ovarian cancer, 2025 has delivered transformative results. Simultaneously, the refinement of biomarker-directed strategies in endometrial cancer and the exploration of novel therapeutic mechanisms, such as glucocorticoid receptor modulation and next-generation antibody-drug conjugates, have expanded the therapeutic arsenal. Furthermore, important strides in surgical de-escalation underscore a parallel evolution toward reducing treatment-related morbidity without compromising oncologic outcomes.

Despite this wealth of new data, the rapid and simultaneous presentation of these studies across multiple venues presents a challenge for clinicians, researchers, and policymakers seeking to synthesize and critically appraise this evidence to inform practice and research directions. There exists a clear need for a comprehensive, systematic evaluation that integrates conference-derived data with peer-reviewed publications, assesses methodological quality, and contextualizes findings within the evolving therapeutic landscape.

This systematic review, therefore, aims to provide a rigorous synthesis and critical appraisal of the most significant therapeutic advances in gynecologic oncology reported throughout 2025. We employed inclusive criteria to capture evidence across the therapeutic development spectrum, from early-phase proof-of-concept studies to confirmatory phase III randomized controlled trials. By systematically integrating data from both major conference presentations and full-length publications, this review offers a timely, consolidated, and evidence-based overview of the current state of the art. It is structured to guide stakeholders by detailing novel therapeutic strategies, elucidating the increasingly central role of biomarkers in clinical decision-making, discussing implications for personalized patient care across different disease sites and stages, and identifying key knowledge gaps and future research priorities. Ultimately, this synthesis seeks to translate a year of remarkable scientific progress into a coherent framework for advancing patient care in gynecologic oncology.

## 2. Methods

### 2.1. Protocol and Registration

This systematic review was designed and conducted in strict accordance with the Preferred Reporting Items for Systematic Reviews and Meta-Analyses (PRISMA) 2020 statement to ensure methodological rigor, transparency, and reproducibility. A detailed study protocol outlining the research question, search strategy, eligibility criteria, data extraction methodology, and planned synthesis approach was developed a priori. This protocol was prospectively registered on the PROSPERO international prospective register of systematic reviews (registration number CRD420251270085) to minimize reporting bias and enhance the review’s credibility. Any deviations from the registered protocol occurring during the review process are explicitly noted and justified in the relevant sections below.

### 2.2. Eligibility Criteria

Studies were included or excluded based on the following predefined criteria, designed to capture evidence of novel therapeutic interventions across gynecologic malignancies.

Studies were considered for inclusion if they met one of the following designs: (1) Phase II or III randomized controlled trials (RCTs); (2) Non-randomized studies (including phase I and II trials) with historical or external control arms; or (3) Phase I and II trials without control arms (single-arm studies).

This inclusive approach was chosen to comprehensively capture all emerging therapeutic strategies, from early-phase proof-of-concept studies to confirmatory phase III trials. The population of interest was adult patients (aged 18 years or older) with a histologically confirmed diagnosis of a primary gynecologic malignancy, including ovarian (including fallopian tube and primary peritoneal cancers), endometrial, cervical, vulvar, or vaginal cancer. Studies of mixed populations were included only if results were reported separately for the relevant gynecologic cancer cohort (Table 1).

The intervention involved the evaluation of a novel therapeutic agent, combination regimen, or treatment strategy. “Novel” was defined as involving a drug or strategy not yet established in the specific clinical setting under investigation by international guidelines at the start of 2025. Included studies were required to report at least one primary efficacy endpoint of overall survival (OS), progression-free survival (PFS), or objective response rate (ORR). For studies without control arms, emphasis was placed on ORR and safety data as primary indicators of activity (Table 1).

The source of the evidence was also a key criterion. The study results were eligible if they were either (a) presented as an oral presentation (including late-breaking abstracts) at the ASCO 2025 Annual Meeting or the ESMO Congress 2025 or (b) published in a peer-reviewed medical or oncology journal with a publication date within the 2025 calendar year. Finally, studies needed to be published or presented in English.

Studies were excluded based on several factors. We excluded studies that did not report key efficacy endpoints (OS, PFS, or ORR) with sufficient data for analysis. Duplicate publications or multiple reports from the same trial cohort were identified, and in such cases, the most comprehensive, recent, or final report (e.g., full publication over abstract) was selected. We also excluded preclinical studies, in vitro investigations, retrospective observational studies, cost-effectiveness analyses, qualitative studies, and reviews. Lastly, studies focused exclusively on supportive care, imaging techniques, surgical techniques without oncologic outcomes, or diagnostic biomarkers not linked to a therapeutic intervention were not included.

### 2.3. Information Sources and Search Strategy

A comprehensive, multi-source search strategy was employed between 1 December and 20 December 2025 to minimize the risk of missing relevant studies.

#### 2.3.1. Conference Proceedings

The official online abstract libraries and virtual meeting platforms for the ASCO 2025 Annual Meeting (meeting library on meetings.asco.org) and the ESMO Congress 2025 (abstract database on oncologypro.esmo.org) were systematically searched. All abstract categories, including Late-Breaking Abstracts, were reviewed to capture all relevant presentations from these premier oncology conferences.

#### 2.3.2. Electronic Databases

PubMed/MEDLINE and Embase were searched from 1 January 2025 to 15 December 2025. Search strings were developed with the assistance of a medical librarian and tailored to each database. They combined Medical Subject Headings (MeSH) and free-text keywords related to four key concepts: (1) Gynecologic cancers (e.g., “Ovarian Neoplasms,” “Endometrial Neoplasms,” “Uterine Cervical Neoplasms,” “Vulvar Neoplasms”); (2) Study design (e.g., “Randomized Controlled Trial,” “Phase II,” “Phase III”); (3) Conferences (e.g., “American Society of Clinical Oncology,” “European Society for Medical Oncology,” “ASCO,” “ESMO,” “2025”); and (4) Therapeutic modalities (e.g., “Immunotherapy,” “Immune Checkpoint Inhibitors,” “Antibody-Drug Conjugates,” “PARP Inhibitors,” “Molecular Targeted Therapy”). Boolean operators (AND, OR) were used to combine these concepts effectively.

#### 2.3.3. Hand-Searching and Grey Literature

To ensure thoroughness, several additional sources were manually reviewed. The tables of contents of leading general medical and specialty oncology journals published in 2025 were examined. These included: New England Journal of Medicine, The Lancet, Journal of Clinical Oncology, JAMA Oncology, The Lancet Oncology, Annals of Oncology, Nature Medicine, Gynecologic Oncology, International Journal of Gynecological Cancer, and Clinical Cancer Research. Furthermore, clinical trial registries, specifically ClinicalTrials.gov and the EU Clinical Trials Register, were searched for completed phase II/III trials in gynecologic cancers that had results posted or updated in 2025. Finally, the reference lists of all included studies and relevant review articles were screened to identify any additional eligible studies that may not have been captured by the primary database and conference searches.

### 2.4. Study Selection Process

Study selection was performed independently by two reviewers (Prof. Nabil Ismaili and Prof. Sanaa El Majjaoui), the latter being formally listed as a co-investigator on the PROSPERO-registered protocol (CRD420251270085). Any disagreements were resolved through discussion; if consensus could not be reached, a third senior reviewer with expertise in gynecologic oncology and systematic review methodology was consulted for final adjudication.

#### 2.4.1. Title and Abstract Screening

All records retrieved from the searches were imported into Covidence systematic review software, where duplicates were automatically and manually removed. The two reviewers independently screened the titles and abstracts of all unique records against the eligibility criteria. Records that clearly did not meet criteria (e.g., preclinical study, wrong cancer type) were excluded. All potentially relevant records or those where eligibility was uncertain based on the abstract proceeded to full-text review.

#### 2.4.2. Full-Text Review

The full-text articles, conference presentation slides, and/or detailed abstract supplements for all records passing the initial screen were retrieved. Reviewers independently assessed these documents against the full eligibility criteria. Reasons for exclusion at this stage were documented for each record (see PRISMA Follow Summary, Table 2). The process was piloted on a random sample of 10 records to ensure consistent application of criteria between reviewers.

### 2.5. Data Extraction and Management

Data from included studies were extracted independently by the two reviewers using a standardized, piloted electronic data extraction form developed in Microsoft Excel.

The extracted data encompassed several key domains. Study characteristics included the trial name or identifier, ClinicalTrials.gov registration number, first author or presenter, year of presentation or publication, journal or conference name, study phase, design, primary endpoint(s), statistical design, and duration of follow-up. Participant characteristics covered the total sample size, number of patients per arm, median age, disease stage or setting, histology, number and type of prior lines of therapy, and key biomarker statuses such as PD-L1 expression, mismatch repair and microsatellite instability status, homologous recombination deficiency status, BRCA mutation status, and other specific genetic alterations. Intervention details involved a detailed description of the experimental and control regimens, including drug names, doses, schedules, route of administration, treatment duration, and permitted supportive care. Outcome data focused on primary outcome results like hazard ratios with confidence intervals and *p*-values for overall and PFS, median survival times, as well as secondary efficacy and safety outcomes including ORR, duration of response, and adverse event profiles. Other relevant data captured key stratification factors, prespecified subgroup analyses, statistical analysis plan details, source of funding, and declared conflicts of interest.

Following independent extraction, the two reviewers compared their completed forms. Any discrepancies were resolved by referring back to the original source document and through discussion to reach a consensus. The final agreed-upon dataset was then used for the subsequent synthesis.

### 2.6. Risk of Bias Assessment Strategy

The methodological quality and risk of bias for each included randomized controlled trial were assessed independently by the two reviewers using the revised Cochrane Risk of Bias tool for randomized trials (RoB 2, version 2019). This tool evaluates five key domains: bias arising from the randomization process; bias due to deviations from the intended interventions; bias due to missing outcome data; bias in the measurement of the outcome; and bias in the selection of the reported result. For each domain, reviewers made a judgment of “Low risk,” “Some concerns,” or “High risk” of bias based on a series of signaling questions, and an overall risk-of-bias judgment was then derived for each study.

For studies initially reported as conference abstracts, the assessment was based on the methodological details provided in the abstract and any accompanying presentation slides or supplemental materials. Where these details were insufficient to judge a domain definitively, it was conservatively rated as having “Some concerns.” Any disagreements between the reviewers in their RoB 2 assessments were resolved through discussion. The results of this risk of bias assessment are summarized in both tabular and graphical form in the results section.

### 2.7. Data Synthesis

Given the substantial clinical, methodological, and statistical heterogeneity across the included studies, which included RCTs and single-arm phase I/II trials, a formal quantitative meta-analysis was not performed. Instead, a comprehensive structured narrative synthesis was conducted to integrate findings from these diverse study designs, acknowledging their respective methodological strengths and limitations.

This process began with a description of studies, including a tabular and narrative summary of their characteristics, populations, interventions, and outcomes. Studies were categorized by design: (1) RCTs and (2) Non-randomized controlled studies. The findings were then organized thematically by primary disease site (cervical, ovarian, endometrial, vulvar cancers), and within each disease site, by clinical setting and therapeutic modality.

For each major trial or group of trials, the synthesis describes the rationale and study design; key efficacy results; important subgroup analyses based on biomarkers; key safety findings; and the authors’ main conclusions. The critical appraisal incorporates appropriate quality assessments: Cochrane RoB 2 for RCTs, discussion of limitations for non-randomized controlled studies (e.g., potential selection bias in historical controls), and acknowledgment of the exploratory nature of single-arm trials. The level of evidence is clearly indicated for each finding.

The synthesis aims to identify overarching themes, concordances, and discordances across studies, particularly regarding biomarker utility, sequencing of therapies, and emerging mechanisms of resistance. All statistical results are reported as presented in the source materials.

## 3. Results

### 3.1. Study Selection

**Figure 1:**
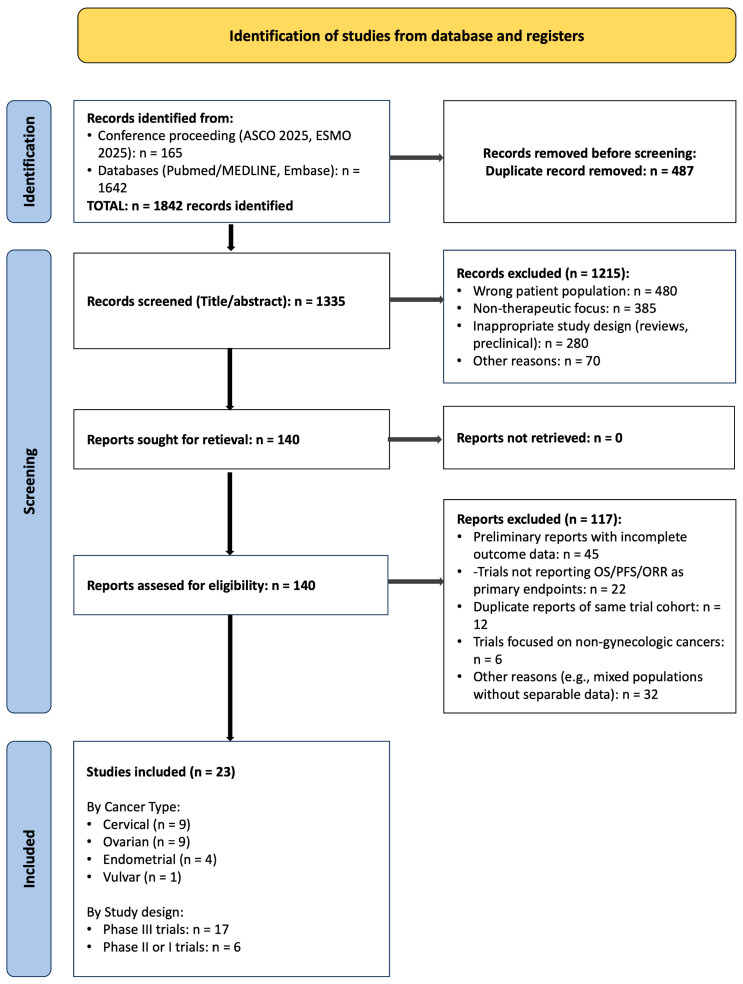
PRISMA 2020 flow diagram of study selection for systematic review of gynecologic oncology advances in 2025. This diagram illustrates the identification, screening, eligibility assessment, and inclusion process for studies. A total of 1842 records were identified from multiple sources. After duplicate removal and screening, 23 studies met inclusion criteria, encompassing RCTs, and single-arm phase I/II trials. The distribution by cancer type and study design is shown.

The study selection process is detailed in the PRISMA 2020 flow diagram (Figure 1). Initial database searches, conference proceedings review, and hand-searching yielded 1842 records. After removal of 487 duplicates, 1355 unique records underwent title and abstract screening. Of these, 1215 records were excluded for not meeting eligibility criteria, primarily due to wrong patient population, non-therapeutic focus (e.g., biomarker discovery, quality of life), or inappropriate study design (e.g., reviews, preclinical studies).

The full-text reports of the remaining 140 records were retrieved and assessed against the eligibility criteria. A total of 117 records were excluded at this stage for the following reasons: preliminary reports with incomplete outcome data (*n *= 45); trials not reporting OS, PFS, or ORR as primary endpoints (*n* = 22); duplicate reports of the same trial cohort where a more comprehensive report was selected (*n* = 12); trials focused on non-gynecologic cancers or mixed populations without separable data (*n* = 6); and other reasons including failure to meet specific population or intervention criteria (*n* = 32).

Ultimately, 23 studies met all inclusion criteria and were included in the qualitative synthesis. These encompassed 17 phase III randomized controlled trials (RCTs), 2 phase II randomized trials, and 4 single-arm phase I/II trials. The distribution by primary disease site was: cervical cancer (*n* = 9, 39%), ovarian cancer (*n* = 9, 39%), endometrial cancer (*n* = 4, 17.5%), and vulvar cancer (*n* = 1, 4.5%).

### 3.2. Study Characteristics

The characteristics of the 23 included studies are summarized in Table 3. The trials collectively enrolled over 12,000 patients worldwide and addressed a wide spectrum of clinical scenarios, ranging from curative-intent treatment for locally advanced disease to the management of heavily pretreated, platinum-resistant recurrences. The 23 included studies comprised: 17 phase-III RCTs, and 6 non-Phase III RCTs (including phase I and II trials, some randomized and others single-arm). The designs of these studies were heterogeneous, including double-blind placebo-controlled trials, open-label RCTs, and single-arm phase I/II trials.

The most common primary endpoint was progression-free survival (PFS), assessed either alone (*n* = 6) or in combination with OS (*n* = 5). OS was a primary endpoint, either alone (*n* = 4) or in combination with PFS (*n* = 5). Other primary endpoints included ORR (*n* = 4), disease-free survival (DFS, *n* = 1), and immunogenicity (*n* = 2). The interventions reflected a significant diversification of the therapeutic arsenal. Immune checkpoint inhibitors (anti-PD-1/PD-L1) formed the backbone of most combination strategies, being evaluated with chemotherapy, chemoradiation, or other targeted agents in the majority of studies (*n* = 13). Other novel strategies included antibody-drug conjugates (ADCs, *n* = 1), PARP inhibitors in maintenance or combination settings (*n* = 2, including one combined with an ICI), and a range of other targeted agents such as glucocorticoid receptor modulators, kinase inhibitors, and anti-EGFR antibodies (*n* = 3). Furthermore, studies investigated chemotherapy optimization or surgical de-escalation (*n* = 2), as well as novel HPV vaccination strategies (*n* = 2).

A defining feature of the 2025 trials was the sophisticated integration of biomarkers. Eleven trials required biomarker testing for enrollment, such as PD-L1 positivity or mismatch repair deficiency/microsatellite instability-high status. All other trials performed extensive biomarker-stratified or exploratory analyses. Key biomarkers evaluated included PD-L1 expression by Combined Positive Score (in cervical and ovarian cancers), mismatch repair and microsatellite instability status (in endometrial cancer), homologous recombination deficiency status (in ovarian cancer), and BRCA mutation status (in ovarian cancer).

### 3.3. Risk of Bias Assessment

**Figure 2:**
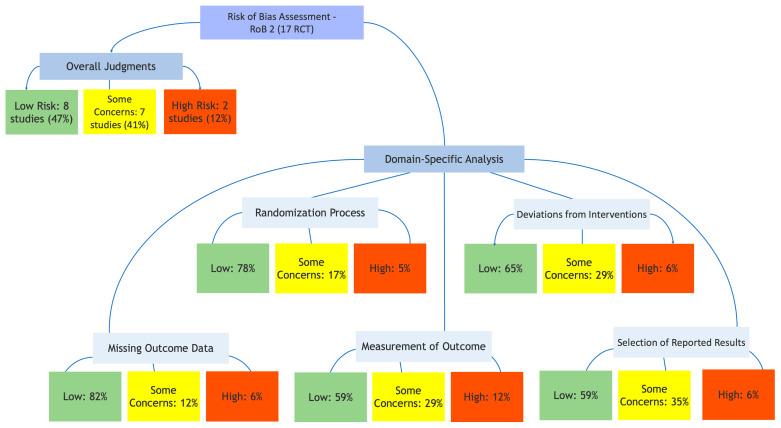
Risk of Bias Assessment for the 17 phase-III Randomized Controlled Trials (RoB 2). Risk of bias assessment using the Cochrane RoB 2 tool for the 17 phase-III RCTs included in the systematic review. Overall, 8 studies (47%) were judged as low risk, 7 (41%) with some concerns, and 2 (12%) as high risk. Domain-specific analysis shows the distribution of risk of bias judgments across the five RoB 2 domains. Color coding: green = low risk, yellow = some concerns, red = high risk.

A differentiated approach was used to assess the methodological quality of included studies. For the 17 phase-III randomized controlled trials (RCTs), the revised Cochrane Risk of Bias tool for randomized trials (RoB 2) was employed. The six non-Phase III-RCTs (including phase II randomized and single-arm trials) were not assessed with RoB 2, as this tool is specifically designed for RCTs. Instead, their methodological limitations, including the absence of randomization, potential selection biases (for studies with historical controls), and the exploratory nature of single-arm designs, are discussed narratively in the synthesis and considered when interpreting findings. The results of the RoB 2 assessment for the 17 phase-III RCTs are presented in Figure 2.

Among these 17 phase-III RCTs, the overall risk of bias was judged as “Low” for 8 studies (47%), “Some concerns” for 7 studies (41%), and “High” for 2 studies (12%).

The eight studies with a low risk of bias were predominantly large, double-blind, placebo-controlled trials, such as ENGOT-ov65/KEYNOTE-B96 and RUBY. These studies were characterized by rigorous methodological features, including clear descriptions of randomization and allocation concealment procedures, low dropout rates, adherence to pre-specified analysis plans, and the use of blinded independent central review (BICR) for outcome assessment.

The seven studies with some concerns were primarily open-label trials where bias in the measurement of PFS was a potential issue, despite the frequent use of BICR to mitigate this risk. For several studies initially reported as conference abstracts, concerns arose in the domain of selection of the reported result due to incomplete reporting of the analysis plan or the conduct of multiple interim analyses.

The two studies judged to have a high risk of bias were limited by their open-label design, the use of investigator-assessed PFS as an endpoint, and a high rate of crossover from the control arm. These factors collectively introduced concerns across multiple domains of the bias assessment.

Overall, the body of evidence from RCTs was judged to be of moderate to high quality. Findings from non-randomized, phase II, and single-arm studies are interpreted with appropriate caution given their inherent methodological limitations while recognizing their value in identifying promising early signals for novel therapies.

### 3.4. Thematic Synthesis of Findings

**Figure 3:**
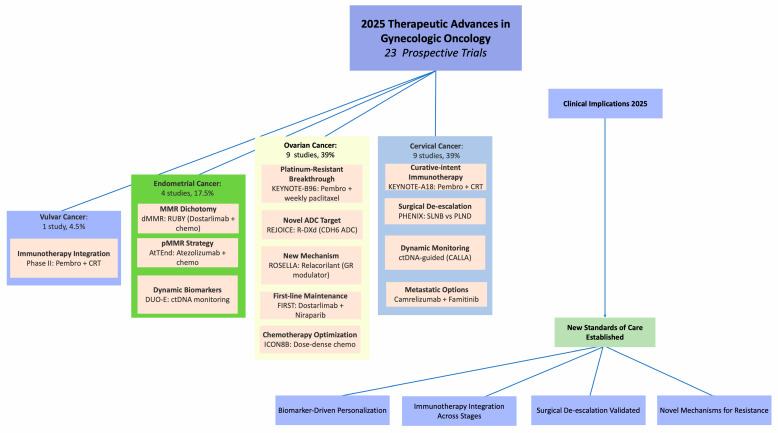
Distribution of 2025 Gynecologic Cancer Breakthroughs: From Biomarker Discovery to Clinical Practice: A visual summary of key findings from a systematic review of 23 prospective trials (17 phase-III RCTs, 6 non-phase-III-RCTs), organized by disease site (cervical, ovarian, endometrial, vulvar cancer) and illustrating major trials and their clinical implications. Abbreviations: Pembro = pembrolizumab; CRT = chemoradiotherapy; SLNB = sentinel lymph node biopsy; PLND = pelvic lymph node dissection; ctDNA = circulating tumor DNA; dMMR/pMMR = deficient/proficient mismatch repair; ADC = antibody-drug conjugate; R-DXd = raludotatug deruxtecan; CDH6 = cadherin-6; GR = glucocorticoid receptor.

Figure 3 provides a visual synthesis of the 2025 therapeutic advances across gynecologic cancers, illustrating the distribution of studies and key breakthroughs by cancer type. This graphical overview highlights the concentration of innovations in cervical and ovarian cancers, which together represent 78% of the included trials. Detailed efficacy and safety outcomes for the major practice-changing trials discussed in this section are summarized in Table 4. The following sections detail these advances organized by disease site, with reference to the corresponding elements in Figure 3 and Table 4.

#### 3.4.1. Cervical Cancer: Expanding the Reach of Immunotherapy and Validating Surgical De-Escalation

Cervical cancer research in 2025 was characterized by two major themes: the successful integration of immunotherapy into the curative-intent, locally advanced setting, and the continued refinement of treatment approaches through biomarker insights and surgical optimization.

Immunotherapy in Locally Advanced Disease: Establishing a New Standard.

The most impactful result came from the final analysis of the KEYNOTE-A18/ENGOT-cx11/GOG-3047 trial, with initial results presented at ESMO 2023 and the final analysis presented at ASCO 2025 [2]. This global, double-blind, phase III trial randomized 1060 patients with newly diagnosed, high-risk, locally advanced cervical cancer (FIGO 2014 stages IB2-IIB with node-positive disease or III-IVA) to receive standard cisplatin-based chemoradiotherapy (CRT) plus either pembrolizumab (200 mg every 3 weeks) or placebo for 15 cycles. With a median follow-up of 39.2 months, the addition of pembrolizumab significantly improved the primary endpoint of PFS (HR 0.70; 95% CI 0.55–0.89; *p* = 0.002). The 24-month PFS rates were 67.8% vs. 57.3% in favor of the pembrolizumab arm. The addition of pembrolizumab also significantly improved OS (36-month OS: 81.6% vs. 74.8%; HR 0.73; 95% CI 0.50–0.90; *p* = 0.004), reaching the prespecified statistical boundary (*p* < 0.04). The benefit was consistent across subgroups, including PD-L1 status (CPS ≥ 1 and <1). The safety profile was manageable, with increased immune-mediated AEs but no new safety signals. This trial definitively establishes pembrolizumab plus CRT as a new global standard of care for this patient population, extending the success of immunotherapy from palliative to curative-intent therapy.

In contrast, the final analysis of the phase III CALLA trial (durvalumab plus CRT), also presented at ASCO 2025, did not meet its primary endpoint of improved PFS (HR 0.91; 95% CI 0.65–1.08; *p* = 0.17) [3]. Despite this negative result, the trial’s extensive translational research program yielded critical insights. Pre-planned analysis of circulating tumor DNA (ctDNA) in 85% of participants showed that 99% had detectable ctDNA at baseline. More importantly, ctDNA clearance during treatment (by week 6) was strongly prognostic, with patients achieving clearance having significantly superior PFS and OS regardless of treatment arm [3]. This work, published in Annals of Oncology, positions ctDNA dynamics as a powerful real-time biomarker for response assessment and risk stratification, potentially guiding future adjuvant therapy decisions [2].

Novel Strategies in Recurrent/Metastatic Disease.

In the recurrent or metastatic (R/M) setting, significant progress was reported with novel combinations. The phase III study by Wu et al. investigated camrelizumab (anti-PD-1) plus famitinib (a multi-targeted tyrosine kinase inhibitor) versus platinum-based chemotherapy as first-line therapy for R/M cervical cancer [4]. Presented at ESMO 2025, the combination demonstrated superior PFS (median 11.3 vs. 7.7 months; HR 0.68; 95% CI 0.49–0.79; *p* < 0.0001) and OS (median 34.4 vs. 23.4 months; HR 0.65; 95% CI 0.49–0.86), offering a potential chemotherapy-free frontline option, particularly relevant for patients unfit for or resistant to platinum.

The phase II COMPASSION-16 trial explored cadonilimab (a PD-1/CTLA-4 bispecific antibody) plus platinum-based chemotherapy with or without bevacizumab. Updated subgroup analyses presented at ASCO 2025 confirmed robust efficacy across various high-risk subgroups, including patients with liver metastases and those with squamous cell carcinoma, reinforcing the potential of bispecific antibodies in this disease [5].

Furthermore, the final OS analysis of the EMPOWER-Cervical 1 trial (cemiplimab vs. chemotherapy in recurrent cervical cancer) was published, confirming the durable survival benefit of cemiplimab in the second-line setting, with a manageable long-term safety profile [6].

Other notable studies included a trial evaluating nimotuzumab (an anti-EGFR antibody) combined with chemotherapy in first-line treatment for advanced cervical squamous cell carcinoma, showing promising efficacy signals [7], underscoring the exploration of diverse targeted pathways.

Surgical De-escalation: Reducing Morbidity Without Compromising Outcomes.

The PHENIX/NCIC-CTG CX.9 trial, presented at the ASCO 2025, addressed a long-standing question in surgical management [7]. This non-inferiority, phase III trial enrolled 1200 patients, with 908 included in the primary analysis, with early-stage cervical cancer (FIGO 2018 IA1 with LVSI–IB2) scheduled for radical hysterectomy. Patients were randomized to comprehensive pelvic lymphadenectomy (PLND) or sentinel lymph node (SLN) biopsy alone (with ultrastaging). If the SLN was negative, no further lymphadenectomy was performed. The primary endpoint was 3-year DFS.

With median follow-up of 42 months, DFS was non-inferior for SLN biopsy versus PLND (HR 0.61; 95% CI 0.33–1.14). The 3-year DFS rates were 91.5% vs. 90.8% (difference 0.7%; 95% CI -2.1% to 3.5%; non-inferiority margin 4%) [8]. Crucially, the rate of significant lower-limb lymphedema (grade ≥ 2) at 24 months was 3.2% in the SLN arm versus 18.7% in the PLND arm (*p* < 0.0001) [8]. This practice-changing study provides level I evidence that SLN biopsy can safely replace systematic PLND in early-stage cervical cancer, significantly reducing chronic surgical morbidity.

Advances in HPV Vaccination.

While not therapeutic interventions for established cancer, two pivotal studies on next-generation HPV vaccines were published in 2025, representing critical advances in primary prevention.

Agbenyega et al. reported in *The Lancet Infectious Diseases* that a single dose of an E. coli-produced bivalent HPV vaccine was non-inferior to the standard two-dose regimen in girls and boys aged 9–14 years, which could dramatically simplify vaccination programs and improve coverage [9]. Additionally, Wang et al. presented phase 2 trial results for an E. coli-produced 9-valent HPV vaccine in Chinese women aged 18–45 years, showing robust immunogenicity and a favorable safety profile, potentially expanding protection against more HPV types with a more scalable production platform [10].

#### 3.4.2. Ovarian Cancer: Breaking the Immunotherapy Barrier and Unleashing Novel Mechanisms

Ovarian cancer research in 2025 delivered arguably the most paradigm-shifting advance of the year in gynecologic oncology: a definitive survival benefit for immunotherapy in platinum-resistant disease. Coupled with breakthroughs in novel therapeutic classes, the outlook for this challenging malignancy has improved substantially.

A Landmark in Platinum-Resistant Recurrent Ovarian Cancer (PRROC).

The ENGOT-ov65/KEYNOTE-B96 trial, presented as a Plenary Session at ESMO 2025, is a landmark achievement [11]. This global, double-blind, phase III trial evaluated pembrolizumab plus weekly paclitaxel (with optional bevacizumab at investigator’s discretion) versus placebo plus weekly paclitaxel (± bevacizumab) in patients with PRROC who had received 1–2 prior lines of therapy. The dual primary endpoints were PFS and OS in patients with PD-L1 CPS ≥ 1 (approximately 75% of the 643-patient ITT population). At the second pre-specified interim analysis (data cut-off March 5, 2025; median follow-up 26.6 months), the trial met both endpoints. In the PD-L1 CPS ≥ 1 population, pembrolizumab significantly improved PFS (median 8.3 vs. 7.2 months; HR 0.72; *p* = 0.0014) and, critically, OS (median 18.2 vs. 14.0 months; HR 0.76; 95% CI 0.62–0.93; *p* = 0.0053). This represents the first statistically significant OS improvement with an immune checkpoint inhibitor-based regimen in ovarian cancer. Benefit was also observed in the ITT population (median OS 17.7 vs. 14 months; HR 0.81; *p* = 0.0114). The safety profile was consistent with known profiles of the individual agents. This trial establishes pembrolizumab + weekly paclitaxel as a new standard of care for PRROC, transforming a treatment landscape once defined by poor outcomes and limited options [11].

Novel Therapeutic Mechanisms Show Potent Activity.

The ROSELLA/ENGOT-ov68 trial, published in *The Lancet*, introduced a novel biological strategy [11,12]. Based on preclinical data implicating glucocorticoid receptor (GR) signaling in chemotherapy resistance, this phase III trial compared nab-paclitaxel combined with relacorilant (a selective GR modulator) to nab-paclitaxel alone in patients with PRROC [12,13]. The combination significantly improved the primary endpoint of PFS per BICR (median 5.7 vs. 3.8 months; HR 0.70; 95% CI 0.54–0.91; *p* < 0.0001) and demonstrated a significant OS benefit at interim analysis (median 15.97 vs. 11.5 months; HR 0.69; 95% CI 0.52–0.92; *p* = 0.012) [13]. This validates GR modulation as a viable therapeutic strategy to overcome chemotherapy resistance.

Antibody-drug conjugates continued to show exceptional promise. The phase II REJOICE-Ovarian01 trial evaluated raludotatug deruxtecan (R-DXd), an ADC targeting cadherin-6 (CDH6), in patients with PRROC. Presented as an ESMO LBA, the primary analysis of the dose-optimization part showed a confirmed ORR of 50.5% in 107 heavily pretreated patients (median 4 prior lines), with a 7.5% complete response rate and a median PFS of 7.8 months. Remarkably, activity was observed across the spectrum of CDH6 expression, suggesting CDH6 is a highly promising target in ovarian cancer. The dose selected for further development was 5.6 mg/kg [14].

Early-phase trials also reported on other novel agents. A phase 1 study of INCB123667, a selective CDK2 inhibitor, showed preliminary efficacy signals and a manageable safety profile in patients with advanced platinum-resistant and refractory ovarian cancer, highlighting another potential target for overcoming resistance [15]. Furthermore, Lee et al. reported results from a phase II trial of pembrolizumab and lenvatinib in recurrent clear cell ovarian carcinoma, a histologic subtype known for its poor prognosis and resistance to chemotherapy, showing encouraging activity that warrants further study [16].

Refining Front-Line and Maintenance Therapy.

Long-term follow-up from the ICON8B trial provided important data on chemotherapy intensity [17]. With a median follow-up of 72 months, this phase III trial in high-risk stage III/IV epithelial ovarian cancer showed that dose-dense weekly paclitaxel + three-weekly carboplatin/bevacizumab significantly improved OS compared to standard three-weekly paclitaxel/carboplatin/bevacizumab (median OS 49.8 vs. 39.6 months; HR 0.79; 95% CI 0.65–0.95; *p* = 0.01) [17]. This reinforces the role of dose-dense chemotherapy within a contemporary bevacizumab-containing regimen for selected high-risk patients.

In the maintenance setting, the FIRST/ENGOT-OV44 trial evaluated the combination of dostarlimab (anti-PD-1) and niraparib (PARP inhibitor) as first-line maintenance therapy in advanced ovarian cancer [18]. Presented as an ASCO LBA, the trial demonstrated a significant improvement in PFS compared to niraparib alone, particularly in homologous recombination-deficient (HRD) positive tumors, suggesting a synergistic effect of combining immunotherapy and PARP inhibition in the upfront maintenance setting [18].

The FZOCUS-1 trial investigated fuzuloparib with or without apatinib (a VEGFR2 inhibitor) as first-line maintenance therapy [18]. Published in *CA: A Cancer Journal for Clinicians*, the study reported that the combination significantly extended PFS compared to placebo, offering a new maintenance strategy, particularly relevant in populations where PARP inhibitor use may be limited by BRCA status or prior bevacizumab [19].

Innovative approaches to delivery were also explored. The OVATION-2 trial provided an updated survival analysis for intraperitoneal IMNN-001 (a TLR8 agonist) in combination with neoadjuvant chemotherapy, showing promising signals for activating the local immune microenvironment in advanced ovarian cancer. The updated analysis showed a median PFS of 14.9 months with IMNN-001 plus NACT versus 11.9 months with NACT alone, and a median OS of 46.0 months versus 33.0 months [20].

#### 3.4.3. Endometrial Cancer: Biomarker Precision Defines the Therapeutic Pathway

Endometrial cancer care in 2025 moved beyond establishing the efficacy of first-line immuno-chemotherapy to refining its application through precise biomarker interpretation, revealing a stark and clinically crucial dichotomy based on mismatch repair status.

Solidifying First-Line Immuno-Chemotherapy, Highlighting Biomarker Dichotomy.

Updated and final analyses from the two pivotal first-line trials provided mature data. The NRG GY018/KEYNOTE-775 update, published in *Nature Medicine*, reported on secondary endpoints with longer follow-up [21]. While OS data remained immature, the PFS benefit of adding pembrolizumab to carboplatin-paclitaxel was sustained in both the mismatch repair-proficient (pMMR) population (HR 0.79) and the mismatch repair-deficient (dMMR) population (HR 0.55). The safety profile remained consistent, without exacerbating chemotherapy toxicity [21].

The publication of the comprehensive dMMR/MSI-H cohort results from the ENGOT-EN6-NSGO/GOG-3031/RUBY trial (dostarlimab) in *Gynecologic Oncology* provided robust evidence of transformative benefit [22]. With a median follow-up of 37.2 months, dostarlimab + chemotherapy demonstrated a striking improvement in PFS versus placebo + chemotherapy (HR 0.28; 95% CI 0.19–0.41) and a clinically meaningful OS benefit (HR 0.32; 95% CI 0.20–0.51; *p* < 0.0001) [22]. The 36-month OS rates were 77% vs. 52%. Responses were highly durable [22].

The most critical biomarker insight came from the final OS analysis of the AtTEnd/ENGOT-EN7 trial (atezolizumab), presented as an ESMO 2025 LBA [23]. While the trial met its primary PFS endpoint in both the overall and dMMR populations, the final OS analysis told a different story. In the dMMR subgroup, atezolizumab + chemotherapy provided a significant OS benefit (HR 0.49; 95% CI 0.28–0.83). However, in the pMMR subgroup, there was no OS benefit (HR 1.02; 95% CI 0.80–1.20) [23]. This clear divergence underscores that for patients with dMMR/MSI-H tumors, first-line immuno-chemotherapy provides a substantial survival advantage, while for those with pMMR tumors, the benefit is primarily a delay in progression without a clear survival impact (based on current data). These mandates universal, rapid MMR/MSI testing at diagnosis to guide first-line treatment selection.

Biomarker Dynamics.

Research continued to explore strategies to improve outcomes, particularly in pMMR disease.

Translational work from the DUO-E trial (durvalumab + chemotherapy) explored longitudinal ctDNA changes [24]. Presented at ASCO, the analysis found that ctDNA clearance after one cycle of therapy (by C7D1→C9D1) was achieved in 48% of patients with pMMR tumors in the durvalumab arm versus 17% in the control arm, and this clearance was a strong early predictor of PFS and OS benefit from the addition of durvalumab, providing another dynamic tool to assess treatment response and potentially tailor therapy duration [24].

#### 3.4.4. Vulvar Cancer: Establishing a New Standard with Immuno-Chemoradiation

Vulvar cancer, a rare and often neglected malignancy, saw meaningful progress in 2025. The phase II study by Yeku et al. provided what may become a practice-changing standard for locally advanced, unresectable disease [25]. This trial evaluated cisplatin-sensitized radiation therapy combined with concurrent pembrolizumab [25]. The primary results, presented at ASCO 2025, demonstrated a high pathologic complete response (pCR) rate of 58% in patients who underwent surgery after chemoradiation-immunotherapy, along with promising 2-year PFS and OS rates that compare favorably to historical controls with chemoradiation alone [25]. The regimen was associated with manageable toxicity, primarily related to radiation. This study provides the first prospective evidence supporting the integration of immunotherapy into the curative-intent management of locally advanced vulvar cancer, mirroring the advances seen in cervical cancer with KEYNOTE-A18.

## 4. Discussion

This systematic review synthesizes the remarkable therapeutic advances across gynecologic oncology that converged in the landmark year of 2025. The evidence, derived from high-quality phase II and III randomized trials presented at major conferences and published in leading journals, reveals several interconnected themes that are collectively reshaping clinical practice: the successful expansion of immunotherapy into new disease settings and stages; the critical and evolving role of biomarkers in guiding therapy; the validation of novel biological mechanisms to overcome resistance; and the importance of treatment de-escalation to reduce morbidity. Below, we discuss these themes by disease site as structured in our results, followed by overarching implications, limitations, and future directions.

### 4.1. Cervical Cancer: From Curative Intent to Dynamic Monitoring and Prevention

The positive result of KEYNOTE-A18 represents the culmination of a decade of research integrating immunotherapy into cervical cancer, successfully moving it from the metastatic (KEYNOTE-826) to the locally advanced, potentially curable setting [2]. This establishes a new global standard, but also raises practical questions about resource allocation, patient selection, and long-term toxicity management in a curative population. The contrasting negative result of CALLA (durvalumab) suggests that not all PD-1/PD-L1 inhibitors are equivalent in this combined modality setting, potentially due to differences in antibody structure, timing of administration, or patient population [3]. However, CALLA’s invaluable contribution lies in its biomarker research. The demonstration that ctDNA clearance is a potent, real-time prognostic biomarker opens the door to risk-adapted strategies [3]. Future trials could use ctDNA persistence post-CRT to select patients for intensified adjuvant therapy (e.g., additional immunotherapy cycles, novel agents), while sparing those with ctDNA clearance from further treatment. This moves beyond static biomarkers (PD-L1) toward dynamic, liquid biopsy-based monitoring.

The expansion of effective options in the recurrent and/or metastatic setting, including chemotherapy-free combinations like camrelizumab-famitinib and bispecific antibodies like cadonilimab, provides much-needed alternatives for patients progressing on or unfit for standard platinum-based regimens [4,6]. The PHENIX trial’s validation of SLN biopsy is a major advance in surgical oncology, directly addressing a significant source of long-term morbidity [8]. Its adoption will improve quality of life for thousands of women annually and represents a model for surgical de-escalation studies in other gynecologic cancers.

Finally, the advances in HPV vaccination with single-dose regimens and novel production platforms represent critical progress in primary prevention, which remains the most powerful long-term strategy for cervical cancer control [9,10].

### 4.2. Ovarian Cancer: Overcoming the Immunotherapy Hurdle and a Therapeutic Renaissance

The success of ENGOT-ov65/KEYNOTE-B96 in PRROC is arguably the most paradigm-shifting finding of 2025 in gynecologic oncology [11]. For years, ovarian cancer was considered “immunologically cold,” and multiple trials of immune checkpoint inhibitors monotherapy or in combination with chemotherapy failed. The key to success likely lies in the rational combination with weekly, metronomic paclitaxel, which has known immunomodulatory properties, and the optional use of bevacizumab, which can normalize tumor vasculature. This regimen appears to convert a “cold” tumor microenvironment into a more responsive one. The statistically significant OS benefit provides a desperately needed new therapeutic pillar and will immediately become the benchmark for future trials in PRROC.

Equally exciting is the emergence of novel, non-immunotherapy mechanisms. The ROSELLA trial validates an entirely new target, the glucocorticoid receptor, implicating stress hormone signaling in chemotherapy resistance [12,13]. Raludotatug deruxtecan’s remarkable activity suggests CDH6 is a highly promising ADC target [14]. These developments signal a therapeutic renaissance for ovarian cancer, offering multiple new lines of effective therapy.

The long-term OS benefit from ICON8B reinforces that dose-dense chemotherapy remains relevant in the era of targeted therapy, particularly for high-risk patients [17]. The FIRST trial’s positive result for dostarlimab-niraparib maintenance creates a new, potentially synergistic upfront option, especially in HRD-positive disease [18]. The exploration of combinations like pembrolizumab-lenvatinib in clear cell carcinoma addresses a specific unmet need within this heterogeneous disease [16].

### 4.3. Endometrial Cancer: The MMR Dichotomy and the Path Forward

The 2025 data crystallizes the treatment algorithm for advanced endometrial cancer: universal MMR/MSI testing is now an absolute imperative. The stark OS dichotomy between dMMR and pMMR tumors, with transformative survival benefits in dMMR (RUBY, AtTEnd) and only PFS benefits without clear OS advantage in pMMR (AtTEnd final analysis), creates a clear two-tier pathway [22,23]. For dMMR/MSI-H tumors, first-line immuno-chemotherapy is the unequivocal standard, offering the chance for long-term remission. For pMMR tumors, the benefit is more nuanced. While immuno-chemotherapy remains a standard option per regulatory labels (based on PFS benefit), the lack of OS benefit invites discussion about alternative strategies or clinical trial enrollment.

This dichotomy presents both a success story for precision medicine and a clear unmet need. Future research must focus on understanding and overcoming resistance in pMMR tumors.

The exploration of quadruplet regimens (combining immuno-chemotherapy with additional targeted agents) and the use of dynamic biomarkers like ctDNA clearance represent promising research directions for improving outcomes in pMMR disease [24].

### 4.4. Vulvar Cancer: A New Curative-Intent Paradigm

The study by Yeku et al., though phase II, is highly practice-informing for this rare cancer [25]. By demonstrating the feasibility and high efficacy of adding pembrolizumab to standard cisplatin-based chemoradiation, it establishes a new potential benchmark for locally advanced, unresectable disease. This mirrors the successful integration pathway seen in cervical cancer and should lead to larger confirmatory studies.

### 4.5. Overarching Implications, Limitations, and Future Directions

#### 4.5.1. Clinical Practice Implications

Guideline committees worldwide will be tasked with rapidly integrating these findings (Figure 3). Key updates will include the adoption of pembrolizumab with chemoradiotherapy for locally advanced cervical cancer [2]; pembrolizumab plus weekly paclitaxel and relacorilant plus nab-paclitaxel for platinum-resistant recurrent ovarian cancer [11,13]; universal mismatch repair testing and first-line therapy with dostarlimab or pembrolizumab combined with chemotherapy for mismatch repair-deficient endometrial cancer [21,22]; sentinel lymph node biopsy for surgical staging in early cervical cancer [8]; and consideration of pembrolizumab with chemoradiation for unresectable vulvar cancer [25].

#### 4.5.2. Critical Appraisal of Limitations

Several important methodological and generalizability limitations warrant careful consideration.

First, the methodological heterogeneity of the included evidence presents a key challenge. While the inclusion of uncontrolled (single-arm) phase I and II trials allowed for a comprehensive capture of early therapeutic signals, this design inherently limits the strength of conclusions regarding comparative efficacy. Results from such studies must be interpreted as preliminary, and the risk of overestimating efficacy, particularly in single-arm trials without randomization or a control group, must be acknowledged. This heterogeneity complicates direct cross-trial comparisons and the ranking of therapeutic strategies.

Second, significant population heterogeneity exists across trials in terms of age, performance status, and comorbidities, potentially limiting the generalizability of findings to real-world populations [26,27]. Third, unequal access to comprehensive biomarker testing (PD-L1, MMR, HRD, ctDNA) across different healthcare settings may create disparities in who can benefit from these advances [28]. Fourth, variable follow-up durations between studies, with some reporting interim analyses while others present final data, complicate cross-trial comparisons and may overestimate effect sizes in early reports [29]. Fifth, the limited generalizability of trial findings primarily from high-income countries to low- and middle-income settings represents a substantial equity concern [30]. Finally, potential publication bias favoring positive results may create an overly optimistic perception of therapeutic efficacy [31].

These limitations underscore that while the inclusive approach provides a complete landscape of innovation in 2025, the highest certainty for practice change remains anchored in the results of large, randomized controlled trials. Findings from early-phase and uncontrolled studies should be viewed as generating hypotheses for validation in confirmatory trials.

#### 4.5.3. Accessibility and Implementation Challenges

The translation of these advances into equitable clinical practice faces substantial barriers. The prohibitive cost of novel immunotherapies and antibody-drug conjugates poses significant challenges for healthcare systems worldwide, particularly in resource-limited settings [32,33]. The requirement for sophisticated molecular testing infrastructure for biomarker assessment creates implementation hurdles in regions with limited laboratory capabilities [34]. Stark North–South disparities in access to innovative therapies exacerbate existing global health inequities [35]. Variable regulatory approval timelines across different regions delay patient access to proven therapies [36]. Additionally, the complexity of treatment sequencing decisions as therapeutic options multiply presents clinical decision-making challenges that require sophisticated guidance [37].

#### 4.5.4. Future Research Priorities

Future research must focus on several key areas informed by these limitations. First, overcoming therapeutic resistance requires understanding mechanisms of primary and acquired resistance to immunotherapy, particularly in mismatch repair-proficient endometrial and ovarian cancers, and to novel antibody-drug conjugates [38,39]. Second, biomarker refinement should move beyond single biomarkers toward integrated genomic and immunologic signatures, while validating dynamic biomarkers such as circulating tumor DNA across disease sites [2,24,40]. Third, addressing treatment sequencing and combinations requires designing intelligent sequential therapy trials and rational combinations, such as immunotherapy with novel antibody-drug conjugates or dual immune checkpoint blockade [41,42]. Fourth, dedicated trials for rare cancers and histologic subtypes are essential, building on progress in vulvar cancer and clear cell ovarian carcinoma [15,25]. Fifth, global health implementation science must develop strategies to improve access to biomarker testing and costly novel therapies in low- and middle-income countries, including integration of simplified vaccination strategies [8,43]. Finally, incorporating patient-reported outcomes and quality of life as primary or co-primary endpoints in more trials will ensure therapeutic advances translate into meaningful patient benefit [44].

## 5. Conclusions

The year 2025 stands as a definitive watershed moment in the field of gynecologic oncology, marking the establishment of new personalized care standards while simultaneously presenting significant implementation challenges. This systematic review consolidates high-level evidence from ASCO, ESMO, and major 2025 publications, revealing multiple, simultaneous, and transformative advances across all gynecologic malignancies. Key breakthroughs include: the integration of immunotherapy into curative-intent management for cervical and vulvar cancers; the unprecedented efficacy of pembrolizumab in platinum-resistant ovarian cancer, breaking a long-standing therapeutic barrier; the solidification of biomarker-defined first-line therapy in endometrial cancer, with clear dichotomy between mismatch repair-deficient and proficient tumors; and the validation of surgical de-escalation strategies in cervical cancer.

Biomarker integration (MMR, PD-L1, HRD, ctDNA) now fundamentally guides therapeutic decisions, while immunotherapy extends its benefit to new clinical contexts across the disease spectrum. Coupled with the emergence of powerful novel therapeutic mechanisms such as glucocorticoid receptor modulators and next-generation antibody-drug conjugates, these developments collectively herald a new era of more effective, personalized, and patient-centered care that offers renewed hope for improved survival and quality of life.

However, these advances present the global oncology community with multifaceted challenges. The heterogeneity of trial populations, methodological limitations of some studies, and persistent global access barriers require adapted implementation strategies. Key challenges include: the rapid and equitable integration of complex, biomarker-driven strategies into diverse healthcare systems; the navigation of escalating treatment costs and resource constraints; and the strategic prioritization of future research to address knowledge gaps, particularly in optimal treatment sequencing and overcoming therapeutic resistance.

Looking forward, research priorities must focus on: developing evidence-based algorithms for treatment sequencing; validating dynamic biomarkers like ctDNA for real-time monitoring; and creating equitable access models to ensure these innovations benefit all patients regardless of geographic or socioeconomic status. Successfully meeting these challenges will be essential to ensure that the remarkable promise of the 2025 breakthroughs is fully realized for all patients affected by gynecologic cancers worldwide, translating scientific progress into tangible improvements in clinical outcomes and quality of life across diverse healthcare settings.

## Figures and Tables

**Table 1:** PICO framework.

Element	Inclusion Criteria
Population	Adult patients (≥18 years) with histologically confirmed gynecologic cancer (ovarian, endometrial, cervical, vulvar, vaginal)
Intervention	Novel therapies (immunotherapy, ADC, PARP inhibitors, targeted therapies, innovative combinations)
Comparison	Standard treatment, placebo, supportive care, historical/external control, or N/A (for single-arm studies)
Outcome	Overall survival, progression-free survival, or objective response rate

**Table 2:** PRISMA Flow Summary.

Step	Number of Studies	Criteria
Database identification	1842	Combined PubMed/Embase/conference searches
Duplicates removed	487	Automatic and manual removal
Titles/abstracts screened	1355	Inclusion criteria applied
Excluded after title/abstract	1215	Wrong population, non-therapeutic
Full-text articles assessed	140	Complete criteria evaluation
Excluded after full-text	117	Incomplete data (*n* = 45), single-arm, Trials not reporting OS/PFS/ORR (*n* = 22), duplicate report (*n* = 12), Wrong cancer type/mixed population (*n* = 6), Other reasons
Studies included	23	All criteria met

**Table 3:** Characteristics of included studies: ASCO/ESMO 2025 presentations and 2025 publications.

Trial/Study Name	Cancer Type	Phase	Design	Population (*n*)	Experimental Arm	Comparator Arm	Primary Endpoint	Data Source
KEYNOTE-A18 [2]	Cervical (LACC)	3	Double-blind RCT	1060	Pembrolizumab + CCRT → maint	Placebo + CCRT → maint	OS, PFS	ASCO 2025
CALLA [3]	Cervical (LACC)	3	RCT, ctDNA-integrated	770	Durvalumab + CCRT	CCRT alone	PFS	ASCO 2025
Camrelizumab + Famitinib [4]	Cervical (R/M)	3	Open-label RCT	443	Camrelizumab + Famitinib	Platinum chemo ± Bev	PFS, OS	ESMO 2025
COMPASSION-16 [5]	Cervical (R/M)	3	RCT, subgroup analysis	445	Cadonilimab + chemo ± Bev	Placebo + chemo ± Bev	PFS, OS	ASCO 2025
EMPOWER-Cervical 1 [6]	Cervical (R/M)	3	RCT	Recurrent cohort	Cemiplimab	Chemotherapy	OS	Published 2025
Nimotuzumab + Chemo [7]	Cervical (R/M)	3	Double-blind RCT	118	Nimotuzumab + chemo	Chemo alone	OS	ASCO 2025
PHENIX [8]	Cervical (Early)	3	Surgical non-inferiority	908	SLNB only	Pelvic lymphadenectomy	DFS	ASCO 2025
HPV Prevention RCT [9]	Cervical (Prev)	3	RCT	Large cohort	Single-dose Cecolin	Two-dose Gardasil	Immunogenicity	Published 2025
E. coli 9vHPV Vaccine [10]	Cervical (Prev)	2	RCT	Chinese women	E. coli 9vHPV vaccine	N/A	Immunogenicity	Published 2025
ENGOT-ov65/KEYNOTE-B96 [11]	Platinum-Resistant Recurrent Ovarian Cancer	3	Randomized, Double-Blind	643 (ITT)	Pembrolizumab + weekly paclitaxel ± bevacizumab	Placebo + weekly paclitaxel ± bevacizumab	PFS per RECIST v1.1 (investigator)	ESMO 2025
ROSELLA [12,13]	Ovarian (PROC)	3	Open-label RCT	381	Relacorilant + Nab-paclitaxel	Nab-paclitaxel alone	PFS, OS	ASCO 2025 and LANCET 2025
REJOICE-Ovarian01 [14]	Ovarian (PROC)	2	Randomized/Dose optimization	107	R-DXd (4.8, 5.6, 6.4 mg/kg)	N/A (dose selection)	ORR	ESMO 2025
INCB123667 [15]	Ovarian (PROC/r)	1	Dose escalation/expansion	90	INCB123667 (CDK2i)	N/A (single-arm)	ORR	ASCO 2025
Pembrolizumab + Lenvatinib [16]	Ovarian (CCOC)	2	Single-arm, two-stage	30	Pembrolizumab + Lenvatinib	N/A (single-arm)	ORR, 6-mo PFS	ASCO 2025
ICON8B [17]	Ovarian (1L High-risk)	3	Three-arm RCT	579	Dose-dense weekly Pac + Carbo + Bev	3-weekly Pac + Carbo + Bev	OS	ESMO 2025
FIRST/ENGOT-OV44 [18]	Ovarian (1L)	3	Double-blind, maint	1138	Dostarlimab + chemo → Dost + Nira	Placebo + chemo → Placebo + Nira	PFS	ASCO 2025
FZOCUS-1 [19]	Ovarian (1L maint)	3	RCT	Chinese cohort	Fuzuloparib + Apatinib	Fuzuloparib alone	PFS	Published 2025
OVATION-2 [20]	Ovarian (Neoadjuvant)	1/2	RCT, immunotherapy combo	112	IMNN-001 + NACT	NACT alone	PFS	ASCO 2025
NRG GY018 Update [21]	Endometrial (Advanced)	3	Double-blind RCT	810	Pembrolizumab + CP → maint	Placebo + CP → maint	OS (secondary)	Published 2025: Nat Med
RUBY/ENGOT-EN6 [22]	Endometrial (dMMR/MSI-H)	3	RCT, biomarker-selected	118	Dostarlimab + CP → maint	Placebo + CP → maint	PFS	Published 2025: Gynecol Oncol
ATEnd/ENGOT-EN7 [23]	Endometrial (Advanced)	3	Biomarker-stratified RCT	549	Atezolizumab + CP → maint	Placebo + CP → maint	OS, PFS	ESMO 2025
DUO-E [24]	Endometrial (Advanced)	3	Three-arm, ctDNA monitoring	718	CP + Durva → Durva ± Ola	CP alone	PFS	ASCO 2025
Cisplatin + Pembro + RT [25]	Vulvar (Unresectable)	2	Single-arm	24	Cisplatin + Pembro + RT	N/A (single-arm)	ORR	ASCO 2025

Abbreviations: RCT—Randomized Controlled Trial; OS—Overall Survival; PFS—Progression-Free Survival; ORR—Objective Response Rate; DFS—Disease-Free Survival; ITT—Intention-To-Treat; PROC—Platinum-Resistant Ovarian Cancer; CCOC—Clear Cell Ovarian Carcinoma; dMMR—Deficient Mismatch Repair; MSI-H—Microsatellite Instability-High; CP—Carboplatin/Paclitaxel; CCRT—Concurrent Chemoradiotherapy; NACT—Neoadjuvant Chemotherapy; maint—Maintenance therapy; Bev—Bevacizumab; Durva—Durvalumab; Ola—Olaparib; R-DXd—Raludotatug Deruxtecan; ctDNA—Circulating Tumor DNA; SLNB—Sentinel Lymph Node Biopsy; HPV—Human Papillomavirus; 1L—First-Line; C7D1→C9D1—Cycle 7 Day 1 to Cycle 9 Day 1 (ctDNA clearance timepoints); ASCO—American Society of Clinical Oncology; ESMO—European Society for Medical Oncology; FIGO—International Federation of Gynecology and Obstetrics; RECIST—Response Evaluation Criteria in Solid Tumors.

**Table 4:** Detailed Outcomes of Major Practice-Changing Trials.

Trial	Population and Setting	Key Efficacy Results	Safety Profile	Biomarker Insights	Clinical Implications
KEYNOTE-A18 [2]	LACC, stage IB2-IVA (*n* = 1060)	PFS HR 0.70 (95% CI 0.55–0.89); OS HR 0.73 (95% CI 0.50–0.90); 36-mo OS: 81.6% vs. 74.8%	Grade ≥ 3 TRAEs: 69.5% vs. 61.5%; No new safety signals	Benefit consistent across PD-L1 subgroups	Pembrolizumab + CCRT new 1L standard
CALLA [3]	LACC (*n* = 770)	Primary PFS negative (HR 0.91, *p* = 0.17); ctDNA: baseline detection 99%, clearance 23% vs. 36% at 6 mo	Manageable profile; No new concerns	ctDNA prognostic: Detectable post-RT → higher recurrence (HR 3.2)	ctDNA for risk stratification; Durvalumab not recommended
Camrelizumab + Famitinib [4]	R/M CC 1L (*n* = 443)	OS HR 0.65 (95% CI 0.49–0.86); mOS: 34.4 vs. 23.4 mo; mPFS: 11.3 vs. 7.7 mo; PFS HR 0.68 (95% CI 0.49–0.79)	Grade ≥ 3: 88.6% vs. 70%; HTN, HFS, proteinuria	Benefit regardless of PD-L1	First effective chemo-free option for R/M CC
PHENIX [8]	Early-stage CC (*n* = 908)	DFS non-inferior (HR 0.61, 95% CI 0.33–1.14); Superior CSS (HR 0.21); No retroperitoneal recurrences in SLNB arm	Significantly reduced morbidity: OR time↓, blood loss↓, lymphedema↓	SLN detection rate > 95%	SLNB new standard for surgical staging
ENGOT-ov65/KEYNOTE-B96 [11]	Platinum-resistant recurrent ovarian cancer (PRROC), 1–2 prior lines (*n* = 643 ITT)	PFS: 8.3 vs. 7.2 mo (HR 0.72; *p* = 0.0014) in PD-L1 CPS ≥ 1; OS: 18.2 vs. 14.0 mo (HR 0.76; 95% CI 0.62–0.93; *p* = 0.0053) in PD-L1 CPS ≥ 1; ITT OS: 17.7 vs. 14 mo (HR 0.81; *p* = 0.0114)	Consistent with known profiles; no new safety signals	Benefit in PD-L1 CPS ≥ 1 population (≈75% of ITT	First statistically significant OS improvement with an ICI in ovarian cancer; new standard of care for PRROC
ROSELLA [12,13]	PROC (*n* = 381)	PFS HR 0.70 (95% CI 0.54–0.91); OS HR 0.69 (95% CI 0.52–0.92); mOS: 15.97 vs. 11.5 months	Anemia 58%, neutropenia 56%, nausea 39%; No new signals	Consistent across subgroups (prior LOT, PFI)	First dual PFS/OS benefit in PROC; New standard
REJOICE-Ovarian01 [14]	PROC (*n* = 107)	ORR 50.5% (95% CI 40.6–60.3); Dose selected: 5.6 mg/kg	Nausea 69%, anemia 57%, asthenia 47%; ILD 3.7%	Responses across CDH6 levels; Higher expression → better outcomes	Promising ADC; Phase 3 planned with 5.6 mg/kg
OVATION-2 [20]	Advanced EOC, neoadjuvant (*n* = 112)	PFS: 14.9 vs. 11.9 months; OS: 46.0 vs. 33.0 months; Benefit in HRD + PARPi maint subgroup	Abdominal pain, nausea, vomiting; No CRS	Enhanced immune activation in peritoneum	Novel intraperitoneal immunotherapy platform
ATEnd/ENGOT-EN7 [23]	Advanced EC (*n* = 549)	ITT: OS HR 0.87 (*p* = 0.082); dMMR: OS HR 0.49 (95% CI 0.28–0.83); pMMR: HR 1.02	No new safety signals	MMR status predictive; PD-L1 not predictive	Atezolizumab + chemo new standard for dMMR EC
DUO-E [24]	Advanced EC (*n* = 718)	PFS benefit with Durva ± Ola; ctDNA clearance: pMMR 48% vs. 17% (C7D1→C9D1)	Olaparib: anemia, fatigue; Durvalumab: immune-related	ctDNA dynamics correlate with outcomes	ctDNA for monitoring; Durva + Ola promising in pMMR

Abbreviation: Population and Setting—Population and Setting; Key Efficacy Results—Key Efficacy Results; Safety Profile—Safety Profile; Biomarker Insights—Biomarker Insights; Clinical Implications—Clinical Implications; LACC—Locally Advanced Cervical Cancer; OS—Overall Survival; HR—Hazard Ratio; CI—Confidence Interval; PFS—Progression-Free Survival; TRAEs—Treatment-Related Adverse Events; PD-L1—Programmed Death-Ligand 1; CCRT—Concurrent Chemoradiotherapy; 1L—First-Line Treatment; ctDNA—Circulating Tumor DNA; RT—Radiotherapy; R/M CC—Recurrent/Metastatic Cervical Cancer; mOS—Median Overall Survival; HTN—Hypertension; HFS—Hand–Foot Syndrome; DFS—Disease-Free Survival; CSS—Cancer-Specific Survival; SLNB—Sentinel Lymph Node Biopsy; PROC—Platinum-Resistant Ovarian Cancer; LOT—Line of Therapy; PFI—Platinum-Free Interval; ORR—Objective Response Rate; mg/kg—Milligrams per Kilogram; ILD—Interstitial Lung Disease; ADC—Antibody-Drug Conjugate; CDH6—Cadherin-6; EOC—Epithelial Ovarian Cancer; HRD—Homologous Recombination Deficiency; PARPi—PARP Inhibitor; CRS—Cytokine Release Syndrome; EC—Endometrial Cancer; ITT—Intention-To-Treat; dMMR—Deficient Mismatch Repair; pMMR—Proficient Mismatch Repair; Durva—Durvalumab; Ola—Olaparib; C7D1→C9D1—Cycle 7 Day 1 to Cycle 9 Day; →—Implique; ↓—lower.

## Data Availability

All data generated or analyzed during this study are included in this published article. The datasets supporting the conclusions of this article are derived from publicly available sources: conference abstracts and presentations from the ASCO 2025 Annual Meeting, ESMO 2025 Congress, as well as published articles in peer-reviewed journals as referenced.

## Abbreviations

ADC	Antibody-Drug Conjugate
AE	Adverse Event
ASCO	American Society of Clinical Oncology
Bev	Bevacizumab
BICR	Blinded Independent Central Review
CC	Cervical Cancer
CCRT	Concurrent Chemoradiotherapy
CDH6	Cadherin-6
CDK2	Cyclin-Dependent Kinase 2
CI	Confidence Interval
CP	Carboplatin/Paclitaxel
CPS	Combined Positive Score
CR	Complete Response
CRS	Cytokine Release Syndrome
CSS	Cancer-Specific Survival
ctDNA	Circulating Tumor DNA
CCOC	Clear Cell Ovarian Carcinoma
dMMR	Deficient Mismatch Repair
DFS	Disease-Free Survival
Durva	Durvalumab
EC	Endometrial Cancer
EGFR	Epidermal Growth Factor Receptor
ESMO	European Society for Medical Oncology
FIGO	International Federation of Gynecology and Obstetrics
GR	Glucocorticoid Receptor
HFS	Hand–Foot Syndrome
HPV	Human Papillomavirus
HR	Hazard Ratio
HRD	Homologous Recombination Deficiency
HTN	Hypertension
ILD	Interstitial Lung Disease
ITT	Intention-To-Treat
LACC	Locally Advanced Cervical Cancer
LOT	Line of Therapy
mDOR	Median Duration of Response
MMR	Mismatch Repair
MSI	Microsatellite Instability
MSI-H	Microsatellite Instability-High
NACT	Neoadjuvant Chemotherapy
Ola	Olaparib
ORR	Objective Response Rate
OS	Overall Survival
PARPi	PARP Inhibitor
PD-L1	Programmed Death-Ligand 1
PFI	Platinum-Free Interval
PFS	Progression-Free Survival
pMMR	Proficient Mismatch Repair
PRISMA	Preferred Reporting Items for Systematic Reviews and Meta-Analyses
PROC	Platinum-Resistant Ovarian Cancer
RCT	Randomized Controlled Trial
R-DXd	Raludotatug Deruxtecan
RECIST	Response Evaluation Criteria in Solid Tumors
R/M	Recurrent/Metastatic
SLNB	Sentinel Lymph Node Biopsy
TKI	Tyrosine Kinase Inhibitor
TLR8	Toll-Like Receptor 8
TRAEs	Treatment-Related Adverse Events
VEGFR2	Vascular Endothelial Growth Factor Receptor 2
1L	First-Line
